# Acute Effects of Multicomponent Training, Resistance Training and Walking on Affect and Enjoyment of Healthy Elderly Individuals: A Randomized Cross-sectional Study

**DOI:** 10.2174/0117450179371185250521110952

**Published:** 2025-05-27

**Authors:** Leonardo Fernandes de Souza, Alberto Souza Sá Filho, Flávia Paes, Vicente Aprigliano, Pedro Augusto Inacio, Sergio Machado

**Affiliations:** 1 Federal University of Santa Maria, Santa Maria, Brazil; 2 Graduate Program at the Evangelical University of Goiás (UniEVANGÉLICA); Anápolis. Postal code: 75083-515, GO, Brazil; 3 Laboratory of Panic and Respiration, Institute of Psychiatry (IPUB), Federal University of Rio de Janeiro (UFRJ), Rio de Janeiro, Brazil; 4 Escuela de Ingeniería de Construcción y Transporte, Pontificia Universidad Católica de Valparaíso, Avda. Brasil 2147, Valparaíso. Postal code: 2362804, Chile; 5 Center of Neuroscience, Neurodiversity Institute, Queimados, Brazil

**Keywords:** Affection, Sarcopenia, Enjoyment, Physical exercise, Health, Preference, Tolerance

## Abstract

**Introduction:**

Physical exercise plays an essential role in muscle function and the emotional well-being of elderly people. These practices potentially contribute to the development of affective response and enjoyment, creating a social and relaxed environment that improves mood and promotes interpersonal connections. Although the affective responses derived from Resistance Training (RT) and Walking Training (WT) are understood, the behavior of these responses, and the enjoyment associated with Multicomponent Training (MCT) remains underexplored, making further investigation warranted.

**Objective:**

The present study aimed to evaluate the acute effect of MCT, RT, and WT on affective responses and enjoyment in elderly women. Additionally, preference and tolerance were also assessed, as well as the established circumplex model of affect.

**Methods:**

Fifteen older women familiar with MCT, RT, and WT participated in the study in 3 visits. The elderly were randomly allocated to a) MCT, b) RT, and c) WT sessions on alternate days. All participants underwent the three modalities, where a single session lasted a maximum of 40 minutes. Each individual answered the Feeling Scale (FS) and Felt Arousal Scale (FAS) immediately before (FS_1_ and FAS_1_), during (FS_2_ and FAS_2_), and immediately after (FS_3_ and FAS_3_). At the end of each training session, individuals also answered the enjoyment scale (PACES) and preference/tolerance questionnaire (PRETIE – Q).

**Results:**

The Friedman test indicated differences only for FS in the face of RT (*p* <0.001) and WT (*p* <0.001), marking a reduction in affect. For MCT, no differences between the three measures performed were observed (*p*=0.513). There were differences in the FS_2_ (*p* = 0.021) and FS_3_ (*p*=0.002) between RT or WT, with no differences for FS_1_ (*p*=0.641). There were differences in the level of body arousal for RT (*p* <0.001), MCT (*p* = 0.021), and WT (*p* <0.001). Differences were observed in FAS_2_ (*p*=0.029) and FAS_3_ (*p*=0.006) between groups, with no differences in FAS_1_ (*p* = 0.314). PACES scale indicated differences between MCT and RT vs. WT (superior). There were no differences between groups for the PRETIE-Q scale for tolerance or preference. The circumplex model admits that the three exercises performed moved into positive domains.

**Discussion:**

Different exercise alternatives are employed with the aim of overcoming barriers relating to adherence to exercise. From this perspective, the plurality of movement patterns and perceptions of pleasure are substantial. Thus, interventions with multimodal characteristics are incorporated as a response to such barriers. Interestingly, our findings on a population of elderly women contrast with the body of literature, as pleasure was not higher for MCT, but rather for WT.

**Conclusion:**

All three exercise modalities elicited positive affective responses. However, only the MCT was able to increase and maintain the affective response until after the exercise ended. WT elicited a higher level of pleasure and enjoyment of physical activity compared to MCT and RT. The circumplex model of affect demonstrated that it remained within positive domains, characterized by a combination of pleasure and energy.

## 1 INTRODUCTION

Physical exercise is widely recognized for its physiological and psychological benefits, particularly in aging populations, where it plays a key role in maintaining muscle function, mobility, and emotional well-being [1, 2]. Among elderly people, adherence to physical activity programs remains a challenge, often influenced by affective responses and perceived enjoyment during exercise [3]. Research suggests that individuals are more likely to maintain an active lifestyle when they associate physical activity with positive affective experiences, reinforcing the importance of exploring how different exercise modalities elicit affective and motivational responses [4, 5].

Affective responses to exercise are primarily assessed through pleasure/displeasure and physiological activation, which can be represented qualitatively through the circumplex model of affect [6]. This framework categorizes affective states based on valence (pleasure/ displeasure) and arousal (activation/ deactivation), providing a multidimensional perspective on emotional responses to physical exercise. However, affective responses are intensity-dependent, as distinct exercise types impose varying physiological demands, cognitive engagement, and environmental conditions that influence perception and enjoyment [2]. Aerobic exercises, such as walking, are often associated with positive affect, particularly when performed at self-selected intensities [7, 8]. On the other hand, Resistance Training (RT) has been reported to elicit mixed affective responses, with lower enjoyment at higher intensities due to increased exertion and neuromuscular fatigue [9–11]. More recently, Multicomponent Training (MCT), which integrates aerobic, strength, balance, and mobility exercises, has been explored as a time-efficient alternative that may optimize both physiological adaptations and affective outcomes [12].

Beyond immediate affective responses, exercise enjoyment is a key predictor of long-term adherence, as it reflects the intrinsic motivation and emotional gratification derived from physical activity [3, 13]. Enjoyment is modulated by the intensity and novelty of exercise, as well as social dynamics, which suggests that group-based activities, such as MCT and Walking Training (WT), may foster greater social engagement and affective benefits compared to solitary exercise formats [14]. Additionally, some individuals naturally prefer higher-intensity sessions, while others show lower tolerance to physiological discomfort associated with more demanding workouts [7, 15]. This interindividual variability underscores the importance of assessing multiple psychological dimensions, rather than solely focusing on affective responses to understand behavioral patterns in older adults better.

Despite growing interest in affective exercise prescription, there remains a lack of research comparing the acute affective responses, enjoyment, and preference/tolerance across different exercise modalities in older adults. While RT and WT have been widely investigated [4, 8, 9], the effects of MCT on affect and enjoyment remain underexplored, particularly in physically active elderly women. Therefore, understanding affective responses and tolerability is crucial for designing exercise programs that promote long-term adherence in the elderly, a population often at risk of sedentary behavior. Furthermore, given the increasing adoption of time-efficient exercise formats in aging populations [16, 17], investigating the psychological impact of MCT is also crucial for optimizing adherence and well-being.

The present study aims to compare the acute effects of MCT, RT, and WT in a group setting on affect, enjoyment, preference, and tolerance in elderly women, using the circumplex model of affect as a conceptual framework. Our primary hypothesis (H1) is that MCT will elicit higher affective valence and enjoyment compared to RT and WT. Our secondary hypothesis (H2) is that no significant differences will be observed in preference and tolerance levels across exercise modalities (MCT, RT, and WT). Additionally, we expect that all three modalities will remain in the positive quadrant of the circumplex model, reinforcing the psychological viability of these interventions for older adults.

## 2 METHODS

### 2.1 Experimental Approach

This research is a quasi-experimental crossover study, which comprises a random division of conditions between the same subjects, who serve as both the treatment and control group. The present study used the assumptions outlined by the “International Committee of Medical Journal Editors” (ICMJE) as a reference, and respected all the recommendations proposed in the “Strengthening the Reporting of Observational Studies in Epidemiology” (STROBE Statement) guideline for reporting parallel group. Individuals were invited to participate in the study through phone calls, and the data collection was carried out at a gym in Santa Maria city located in southern Brazil (Rio Grande do Sul, BR). Data was collected for 4 months throughout the year 2024. Individuals participated in the study voluntarily, and were recruited from the gym’s internal database.

For the data collection, elderly individuals who met the eligibility criteria were initially contacted through phone calls conducted at the training center (gym) and the affiliated university. Due to physical space limitations, initially, up to 20 slots were made available to conduct the MCT intervention, considering potential dropouts. In case of insufficient adherence, an additional 20 slots would have been opened for a second intervention phase.

The selected participants signed the consent form (there was no refusal). Subsequently, the elderly were randomly allocated to a) MCT, b) RT, and c) WT sessions, on alternate days with one-week intervals between each session. All participants underwent the three training modalities in a single session, lasting a maximum of 40 minutes.

To ensure proper understanding and minimize potential biases in responses, a structured familiarization session was conducted 24 hours before each participant’s first intervention. During this session, a trained researcher provided detailed explanations about the scales, their scoring systems, and the interpretation of responses. Participants were guided through practical examples, and asked to verbalize their understanding to ensure comprehension. Additionally, participants were given the opportunity to apply the scales in a simulated exercise scenario to ensure they felt comfortable using them during the actual sessions.

On the day of the session, each individual answered the questionnaire to assess psychological data (Feeling Scale – FS and Felt Arousal Scale – FAS) immediately before (FS_1_ and FAS_1_), during (FS_2_ and FAS_2_), and immediately after (FS_3_ and FAS_3_) the respective training session. At the end of each training session, individuals also answered the Physical Activity Enjoyment Scale (PACES) and Preference for and Tolerance of the Intensity of Exercise Questionnaire (PRETIE – Q).

### 2.2 Sample

The study recruited 15 female individuals, aged between 60 and 75 years. The inclusion criteria for participation in the study included: 1) individuals had to be familiar with RT and WT, 2) have practiced physical exercises for at least 3 months, 3) not have any neuromuscular injuries that could interfere with the performance during the training, and 4) not have any neurological or mental illness. Participants with previously diagnosed cardiovascular diseases or disabling motor and functional deficits were excluded. As a criterion to remain included in the study, participants who did not complete any of the training sessions proposed in the study, as well as those who did not submit the free and informed consent form, were excluded from the project.

Participants performed and remained in the experiment of their own free will, adhering to the informed consent form, and the project was approved by the Ethics Committee for Research with Human Beings (CEP) of the university in accordance with Resolution no. 466/2012 National Health Council, explaining that this research is voluntary and not mandatory and that the information obtained will be kept anonymous.

For the sample calculation, an ANOVA for repeated measures (within-between interaction) was considered, establishing input of the effect size=0.45, alfa α = 0.05; Power (1-β err prob) = 0.80, number of groups = 3; Number of measurements = 3; Correlation among repeated measures = 0.5; designating a sample size of 15 participants (G*Power, Version 3.1.9.4).

### 2.3 Variables and Outcomes

As the primary outcome, the categorical variables of the Feeling Scale (FS) and Felt Arousal Scale (FAS) were assessed, along with the Physical Activity Enjoyment Scale (PACES), to evaluate enjoyment levels. As secondary outcomes, the Preference and Tolerance for the Intensity of Exercise Questionnaire (PRETIE-Q) and the Circumplex Model of Affect were utilized to explore the different aspects associated with the various exercise modalities.

### 2.4 Psychological Instruments

The FS is a bipolar scale of 11 items that measure the immediate response to exercise. It ranges from +5 (very good) to -5 (very bad), with 0 as the neutral value. Positive values ​​represent pleasure, while negative values ​​represent displeasure. The anchor used was “How do you feel now?”. The FAS is a scale that assesses the level of activation perceived by the individual, which consists of 6 points, where 1=low activation and 6=high activation [18, 19]. These scales have been validated for use in exercise-related affective response studies [18, 19]. Additionally, their translation and adaptation to Portuguese have been carried out and validated for use with Brazilian populations [18].

PACES scale consists of 18 items, and each item has two opposite poles (bipolar) that are separated by a 7-point scale (1 = “I like it,” 7 = “I hate it,” and 4 = “neutral”). The values ​​of each item are added together, with a maximum score of 126 points and a minimum of 18 points, resulting in an unidimensional measure of pleasure in physical activity; the higher the score, the greater the pleasure of the activity. This type of scale is based on the importance of language to express feelings. Some items on the scale should have their values ​​reversed [19]. This scale has been widely used in exercise psychology research, and has been translated and validated for Brazilian Portuguese [19].

Finally, the PRETIE-Q consists of two subscales: 1) Preference, which evaluates an individual's tendency to choose and enjoy higher or lower-intensity exercise, and 2) Tolerance, which assesses one's ability and willingness to continue exercising despite high perceived exertion. The questionnaire comprises 16 items, with 8 items corresponding to each subscale. Responses are provided on a 5-point Likert scale, ranging from 1 (strongly disagree) to 5 (strongly agree). Higher scores indicate a greater preference for or tolerance of high-intensity exercise. To ensure accurate responses and comprehension, participants completed the questionnaire in a supervised environment, with a researcher available to clarify any doubts. A brief explanation of the concepts of exercise intensity and tolerance was provided beforehand to ensure a uniform understanding across all participants [20]. This instrument has been validated in different populations [15] and was also translated and adapted for Brazilian Portuguese, demonstrating good psychometric properties [20].

### 2.5 Training Sessions

For the MCT session, movement patterns that were based on bodyweight were used. The session consisted of a ten-minute warm-up period with preparatory exercises, including light joint rotations and active mobility stretches, such as shoulder, hip, ankle, and static. For the conditioning phase, the following components were stimulated: coordination, strengthening of the core, agility, and muscle strength. Table **1** presents the exercises and their sequence and prescriptions, which are similarly described and adapted by Sá Filho *et al.* [21]. The exercises were performed in a circuit format, and the interval was “*ad libitum*” (but the time was adjusted to maintain the total session time). The total conditioning phase was 15 minutes. Finally, a relaxation outcome was performed for 10 minutes.

For RT, the warm-up was carried out on the equipment itself in a self-selected workload (12-15 reps). The conditioning phase followed the recommendations of the American College of Sports Medicine (ACSM) guideline, using only equipment. Multi-joint exercises were prioritized for upper/lower limbs and core, encompassing all major muscle groups. The series lasted approximately 40 minutes in total.

The workload was self-selected by the participants within a range that allowed them to complete the prescribed number of repetitions (10-12 reps per set), while maintaining proper form and control. For this study, the one-repetition maximum (1RM) test was excluded, in order to align with the recommendations for older adults. Additionally, researchers and instructors closely monitored the participants to ensure that the load selection did not compromise exercise execution or increase fatigue beyond the intended intensity. Table **2** presents the exercise selection and prescription.

During the relaxation phase, participants engaged in low-intensity stretching exercises focused on the major muscle groups targeted in the training. This phase lasted approximately 10 minutes and included breathing exercises and static stretching, focusing on areas, such as the quadriceps, hamstrings, calves, shoulders, and back to facilitate post-exercise recovery. The environment was kept calm, and participants were encouraged to focus on deep breathing to enhance relaxation and recovery.

Finally, for WT, an outdoor walk was performed, and the Talk Test was used during the walk to control the intensity [22]. To control intensity at a moderate to high level, subjective effort was suggested. The same exercise time as the RT session was assigned to the WT session.

All sessions were performed at the same time of day. The elderly were monitored by trained professionals to maintain exercise intensity throughout the sessions. A moderate to high rate of perceived exertion was suggested for all exercise sessions.

### 2.6 Randomization

For participant allocation, a simple randomization process was carried out to assign each group to one of the three training modalities: MCT, RT, or WT. Since all participants underwent the three training conditions in separate sessions, randomization was applied to determine the order of the modalities. This approach helped minimize potential order effects. To implement this process, a sealed, opaque envelope method was used. Each participant drew a numbered paper (1, 2, or 3), which corresponded to the first modality they would perform. The second and third sessions were then systematically assigned to complete the sequence. As the sessions were conducted in a group setting, participants were aware of the different training modalities being applied. However, since they were familiar with all three exercise modalities beforehand, their responses were expected to be influenced primarily by the training characteristics rather than by novelty effects.

### 2.7 Data Analysis and Processing

To avoid possible biases in the analysis, the data were collected by a researcher associated with the project and the research group (L.F.S.), and analyzed by a second participant external to the process (A.S). The researcher responsible for data analysis remained blinded throughout the data collection process. The names of all participants remained confidential, being excluded from the technical file and replaced by numbers.

### 2.8 Statistical Analysis

The initial assumptions were made due to the ordinal nature of the categorical variables (FS, FAS, and PACES), and all analyses were conducted using nonparametric statistical methods. General characterization data were expressed as mean and Standard Deviation (SD). The principal data were expressed as median and confidence interval for 95% (CI^95%^). A Friedman test compared repeated measures of the study's dependent variables (FS, FAS), and a Kruskal-Wallis test was then used for the comparison between each group (FS, FAS, PACES, and PRETIE – Q). Finally, the circumplex model was created to visually present the effects of each exercise session in the determined quadrants. The p-value (0.05) was considered for all analyses. GraphPad Prism (version 8.0) was used to construct the results graphically, and SPSS (version 20.0) was used to statistically analyze the data.

## 3 RESULTS

### 3.1 General Information

Study 15 female participants had a mean age of 66.6 ± 3.8 and a mean total exercise experience of 60.8 ± 20.7 months, staying regular and engaged for at least six months. The group of elderly people was previously engaged in a regular exercise program. Hence, no sample was lost throughout the collection process. Participants were properly monitored by telephone or messaging applications after each exercise session, and no deleterious effects were reported. Fig. (**1**) shows the flow of participant entry and exclusion.

### 3.2 Primary Outcome

The Friedman test revealed significant differences only for FS in the face of RT (*p* < 0.001) and WT (*p* < 0.001), suggesting a reduction in the affect of participants. For MCT, no differences were observed between the three measures performed (FS_1_ *vs*. FS_2_ *vs*. FS_3_, *p* = 0.513). The Kruskal-Wallis test demonstrated significant differences in the FS_2_ (H = 7.717; *p* = 0.021) and FS_3_ (H = 12.557; *p* = 0.002) measures between the RT or WT groups, with no differences for FS_1_ (H = 0.889; *p* = 0.641). Fig. (**2**) shows the comparison between moments for FS.

When comparing FAS scores, there were differences in the level of body arousal for RT (*p* < 0.001), MCT (*p* = 0.021), and WT (*p* < 0.001). The Kruskal-Wallis test demonstrated significant differences in FAS_2_ (H = 7.114; *p* = 0.029) and FAS_3_ (H = 10.271, *p* = 0.006) measures between groups, with no differences for FAS_1_ (H = 2.316, *p* = 0.314).

Finally, the analysis of the PACES scale using the Kruskal-Wallis test showed significant differences (MCT: 61.0 - CI^95%^ = 60.3 to 62.8, RT: 61.0 – CI^95%^ = 59.6 to 64.1, WT: 66.0 – CI^95%^ = 63.3 to 68.5. H = 6.634 and *p* = 0.036), suggesting greater enjoyment for the WT session. Fig. (**3**) presents the results of the PACES scale

### 3.3 Secondary Outcome

Kruskal-Wallis analysis did not demonstrate significant differences between groups for the PRETIE-Q scale, both for tolerance (MCT: 26.0 - CI^95%^ = 23.5 to 28.1, RT: 25.0 – CI^95%^ = 22.9 to 27.8, WT: 26.0 – CI^95%^ = 24.5 to 28.6. H = 0.263 and *p* = 0.877), and preference (MCT: 29.0 - CI^95%^ = 26.4 to 30.2, RT: 28.0 – CI^95%^ = 26.4 to 29.1, WT: 28.0 – CI^95%^ = 25.3 to 29.2. H = 0.793 and *p* = 0.673).

Finally, despite the reduction in affect during RT or WT sessions, the circumplex model admits that the three exercises performed moved into positive domains (energy and pleasure), and were maintained during and after the session. This suggests viability in the three exercise conditions for the elderly population. Fig. (**4**) presents the cartesian circumplex model of affect.

### 3.4 Potential Confounding Factors

In analyzing the results of the present study, it is important to highlight certain potential confounding factors that may have influenced the affective responses observed in the baseline. One notable aspect was the elevated baseline scores on the FS across all conditions. Typically, affective responses at baseline tend to present more neutral values [8]; however, in this study, participants exhibited notably high FS scores before the interventions.

ssA possible explanation for this phenomenon lies in the social dynamics of the participants. All individuals were part of a group training program, and were accustomed to exercising together at the same location and time. This pre-existing social interaction likely contributed to a more positive affective state before engaging in the experimental sessions, influencing initial FS scores [23]. While this effect was consistent across all conditions—ensuring a comparable baseline affect—it should be acknowledged as an external influence on the results.

Despite this initial deviation, our primary focus was on the post-exercise FS scores, which reflect changes in affect in response to different training modalities. The results demonstrated that while multicomponent training was able to maintain the affect, both RT and WT led to a decrease in FS scores over time. This reinforces the importance of assessing affective responses dynamically rather than relying solely on baseline measures.

Additionally, other potential confounding variables should be considered, such as individual differences in exercise preference, tolerance, and prior training experience, which may have subtly influenced affective responses. While our sample consisted of physically active older adults with prior exposure to the training modalities, personal inclinations toward specific types of exercise could have shaped the enjoyment and affective outcomes.

To mitigate the impact of these confounding factors, we ensured randomization of the exercise sessions, controlled training intensity, and maintained consistency in session structure across participants. Despite this potential bias, the final outcome was influenced by the types of intervention, as observed in Fig. (**4**), in the circumplex model.

## 4 DISCUSSION

The aim of this study was to compare the acute effects of MCT, RT, and WT on the affective experiences and enjoyment in elderly individuals. As the primary hypothesis (H^1^), we expected to find that multicomponent training generated better levels of affective response and enjoyment in the practitioners, compared to RT and WT. Therefore, our alternative hypothesis was partially accepted, since the enjoyment scores were higher for the walking session.

Firstly, multicomponent training has been proposed as an alternative to traditional RT or aerobic exercise, given that its configuration dynamics supposedly favor metabolic [17, 24] and neural/ muscle adaptations [12, 25], as well as issues with time efficiency [26]. We are aware that time is a barrier to adherence to exercise, which justifies the practice of “exercise snacks” [27]. However, Callado-Mateo *et al.* [27], in their comprehensive umbrella review, identified another important factor associated with exercise abandonment: the homogeneity of sessions, *i.e.*, the lack of exercise variety and the freedom to decide the type of exercise to be performed.

Technically, MCT sessions are designed to explore multiple physical strengths in a variety of ways and shorter amount of time compared to traditional aerobic and RT [28]. Therefore, from the perspective of exercise adherence and stimulus variability, it seems plausible to us that such barriers can be overcome through multicomponent work, which is in line with the previous argument. Despite this rationale, to date, there are no reports on the effects of multicomponent training in psychophysiological responses [29-31]. Furthermore, studies that address the subject do not observe responses in elderly individuals [32], and generally involve sedentary participants [33], making the results difficult to compare. Furthermore, some studies still diverge reasonably from the time-efficient characteristic despite observing interesting metabolic adaptation in responses to MCT. For example, Heubel *et al.* [34], demonstrated the improvement of different physical valences (flexibility, strength, and cardiorespiratory) in 16 weeks of MCT in elderly patients with type II diabetes. Despite the MCT configuration, the time of the training sessions performed in the study (> 60 minutes) differs from that performed in our study.

Similarly, Faro *et al.* [32] used the termed functional training, compared to traditional RT, to determine the acute affective responses and enjoyment of college-aged female participants. Functional training can also involve MCT, combining varied muscular/joint work in different planes of movement. The different types of RT involved 10 repetitions on each side for a total of 160 repetitions (equally). Functional training was based on free or bodyweight movements, such as modified (box) push-ups, goblet squats, dumbbell split-stance, dumbbell 2-leg Romanian deadlift, walking lunge with rotation, dumbbell wood chop, and step-up were used. Both types of exercises increased affective valence positively, immediately and 15 minutes later, and reduced state anxiety. Higher levels of enjoyment were observed after functional training sessions compared to traditional RT. Based on this effect, together with the affective response, it can be assumed that functional training would establish itself as an interesting strategy for regular maintenance of exercise and affect. Interestingly, this study partially diverged from such responses, as the enjoyment was not superior for MCT but rather for WT, potentially due to our sample of elderly women.

Regarding session intensity, many studies demonstrate that light-moderate intensities (40-70% 1RM) of strength exercises presented better affective responses than high intensities (80-100% 1RM) [9-11, 35]. Although we did not investigate exercise sessions at high intensities, our results seem to be in line with what the literature recommends and the possible affective responses. This response is found in a similar way to continuous aerobic exercise, which proposes an analogy with an inverted “U” to guide responses related to affect [4, 36]. Through a meta-analysis, Reed and Ones [37] revealed that low-moderate intensities of aerobic exercises with duration recommended by the ACSM are more effective in inducing activation of positive affect. Additionally, Ekkekakis *et al.* [38] proposed the ventilatory threshold as a sensitive biomarker that is potentially associated with affective changes. Thus, moving from light intensities to around the ventilatory threshold seems to be recommended, especially for elderly populations [39].

According to Ekkekakis [2, 6], the affective response is modulated by the cognitive domain, which occurs predominantly during the maintenance of homeostasis and includes low-moderate exercise intensities, in addition to an interoceptive domain, corresponding to the physiological events triggered by the increase in metabolism. In cases of high intensities, homeostatic control is excessively disturbed, supplanting cognitive interactions. Furthermore, from an evolutionary perspective, the breakdown of homeostasis is perceived as a threat and, for most subjects, generates a negative affective response and the potential discontinuation of the activity [40]. On the other hand, during low-moderate intensities, most subjects perceive the activity as pleasurable, in line with what was observed in this study. However, considering the influence of several cognitive factors (*i.e.*, self-efficacy, self-esteem, and experience with the activity), the responsiveness to the same stimulus is still highly individual [41, 42].

Finally, secondarily, tolerance and preference could be assessed in elderly participants regarding the different types of exercises. We are aware that in young women, preference and tolerance are directly associated with exercise intensity, adequately reflecting the total score of the PRETIE-Q scale [3, 15], and yielding good psychometric properties. Additionally, it was demonstrated that preference and tolerance were positively associated with pleasure (r =0.29, *p* <0.001; r =0.20, *p* <0.05, respectively). The study of Ekkekakis *et al.* [15] indicates that of the 289 women evaluated, 67 women preferred moderate exercise, and 174 women preferred light-intensity exercise (83.3% in total). These responses are in line with what was proposed in this study. However, the three types of exercise sessions did not reveal differences between preference and tolerance scores, which can potentially be explained by the intensity pattern suggested throughout the sessions, and by the intrinsic motivational factors of this group. However, the initial hypothesis that multicomponent training would produce better preference responses was refuted. Despite this, both protocols can be considered in training programs for elderly people.

Additionally, the greater enjoyment reported during the WT session, compared to MCT and RT, may be influenced by psychological and environmental factors. Walking is a familiar and low-complexity activity, which may contribute to a more positive affective response, particularly in older adults. Research indicates that individuals tend to experience greater pleasure when engaging in activities that feel natural and require minimal cognitive or motor effort, leading to higher adherence rates and improved mood regulation [6, 15, 4].

The outdoor setting and social engagement associated with walking may have further enhanced the enjoyment levels observed. Exercising in open spaces has been linked to greater feelings of relaxation and positive emotions, as exposure to nature and fresh air may reduce perceived exertion, and increase psychological well-being [43]. Moreover, group-based physical activities foster a sense of social connection and motivation, which is particularly relevant in elderly populations, as social interactions during exercise have been shown to boost overall enjoyment and emotional engagement [14].

These findings reinforce the notion that affective responses to exercise are not solely determined by physiological effort, but are also shaped by contextual factors, such as environmental setting, social interaction, and familiarity with the activity. Understanding these influences can help guide exercise prescription strategies aimed at optimizing adherence and psychological benefits in older adults.

## 5 LIMITATIONS

Some limitations need to be highlighted. The first pertains to the diversity of variables involved in resistance training, including contraction time, number of sets, repetitions, intensity, recovery time, session duration, and exercise selection. These variables significantly influence affective responses, yet the study prioritized ecological validity over precise intensity control. By doing so, we aimed to mirror real-world training conditions as closely as possible, ensuring that the observed outcomes reflected the typical experiences of elderly individuals in fitness centers. However, this approach inherently limits the ability to standardize and directly compare exercise-induced affective responses. Future research should seek greater precision in controlling these training variables and consider incorporating training impulse (TRIMP) measures to quantify the physiological impact of each exercise modality.

A second limitation relates to the scope of the participant sample, which was composed exclusively of elderly women. While this homogeneity helped control for potential gender-related variability in affective responses, it restricts the generalizability of our findings to male participants or individuals of other genders. Future studies should expand their participant demographics to explore potential differences in how various populations respond to multicomponent, resistance, and walking training.

Additionally, our sample was recruited from a specific gym facility in Southern Brazil, limiting diversity in socioeconomic status, ethnicity, and geographic background. These factors could influence exercise perception, adherence, and affective responses, and future research should strive for a broader representation of different cultural and economic contexts to enhance external validity.

Another challenge was the inherent difficulty in standardizing workload across different exercise modalities. Given that each modality—multicomponent training, resistance training, and walking—has distinct characteristics and physiological demands, achieving direct equivalence in exertion and stimulus was challenging. Although we applied randomization to balance these effects, variations in individual preferences, prior training experiences, and psychological factors may have influenced responses.

Finally, this study was designed to assess acute affective responses without evaluating long-term adherence or behavioral adaptations resulting from continued engagement in these exercise modalities. Future longitudinal studies are needed to investigate how repeated exposure to these training methods influences sustained affective responses, motivation, and adherence over time. Despite these limitations, our findings contribute to the growing body of knowledge on affective responses to exercise in elderly populations.

## CONCLUSION

The findings of this study indicate that all three exercise modalities elicited positive affective responses, reinforcing their potential as viable training strategies for elderly individuals. However, only the MCT was able to increase and maintain the affective response until after the exercise ended. WT elicited a higher level of pleasure and enjoyment of physical activity compared to MCT and RT. Although a reduction in affective valence was observed during RT and WT, the circumplex model of affect demonstrated that it remained within positive domains, characterized by a combination of pleasure and energy. This suggests that, despite fluctuations in immediate affective responses, participants did not transition into negative affective states. Finally, there was no difference between the exercise sessions for tolerance and preference.

## Figures and Tables

**Fig. (1):**
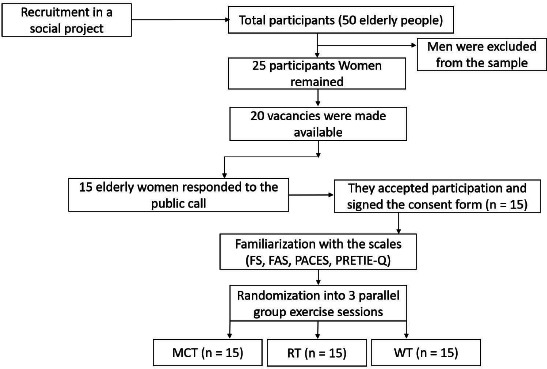
Entry and exclusion of participants.

**Fig. (2):**
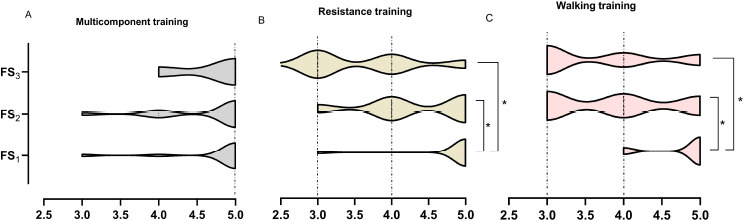
Differences between moments for FS.

**Fig. (3):**
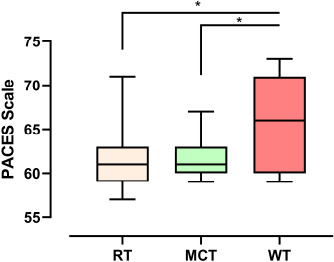
Results of the PACES scale

**Fig. (4):**
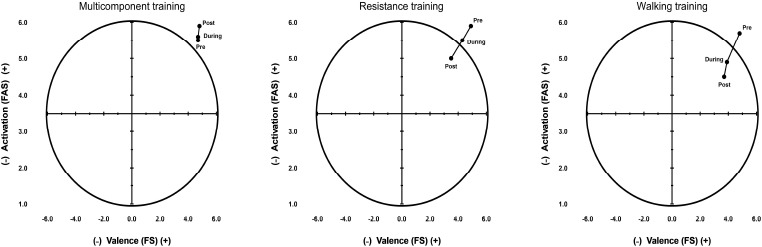
Circumplex model of effect.

**Table 1:** MCT Exercise sequence and prescription.

Category	Sequence	Exercise	Prescription
Upper Body	1	Push up (or adapted)	10-12 reps
	6	Ball throw (or adapted)	
	11	Dips on box	
	15	Shoulder push up (on the ground)	
Lower Body	2	Hip thrust (on the ground)	12-15 reps
	7	Squat or split squat	
	12	Lunge (or walking lunge)	
	16	Calve raises	
	19	Good morning	
Aerobic Demand	3	Jumping jacks	15-30 reps
	8	Jump rope (single or double under)	
	13	Burpees	
	17	Sprawl	
Core	4	Hollow body (or hold)	30 seconds
	9	Arch body (or hold)	
	14	Sit-ups	
	18	Plank or side plank	
Agility and Coordination	5	Single steps (ladder drills)	1 minute
	10	High knees step (ladder drills)	

**Table 2:** Exercise selection and prescription for RT.

Exercise Category	Exercise	Reps	Sets	Rest
Upper Body	Bench press	10-12 reps	3 sets	30-60 s
	Lat pulldown			
	Shoulder press (seated)			
Lower Body	Leg press 45º			
	Leg curl (seated)			
	Leg extension			
	Calf raises (standing)			
Core	crunches			
	Back extension			

## Data Availability

All data generated or analyzed during this study are included in this published article.

## LIST OF ABBREVIATIONS

ACSM: = American College of Sports Medicine
ICMJE: = International Committee of Medical Journal Editors
FS: = Feeling Scale
FAS: = Felt Arousal Scale
MCT: = Multicomponent training
PACES: = Physical Activity Enjoyment Scale
PRETIEQ: = Preference for and Tolerance of the Intensity of Exercise Questionnaire
RT: = Resistance Training
WT: = Walking Training
1RM: = One Repetition Maximum
TRIMP: = Training Impulse

## STANDARDS OF REPORTING

STROBE guidelines were followed.
